# Instant quantification of sugars in milk tablets using near-infrared spectroscopy and chemometric tools

**DOI:** 10.1038/s41598-022-23537-7

**Published:** 2022-11-05

**Authors:** Chanat Thanavanich, Nutthatida Phuangsaijai, Chanidapha Thiraphatchotiphum, Parichat Theanjumpol, Sila Kittiwachana

**Affiliations:** 1 Department of Chemistry, Faculty of Science, Chiang Mai University, Chiang Mai, 50200 Thailand; 2 Postharvest Technology Research Center, Faculty of Agriculture, Chiang Mai University, Chiang Mai, 50200 Thailand; 3 Postharvest Technology Innovation Center, Ministry of Higher Education, Science, Research and Innovation, Bangkok, 10400 Thailand

**Keywords:** Chemistry, Engineering, Mathematics and computing, Optics and photonics

## Abstract

Milk tablets are a popular dairy product in many Asian countries. This research aimed to develop an instant and rapid method for determining sucrose and lactose contents in milk tablets using near-infrared (NIR) spectroscopy. For the quantitative analysis, a training set composed of laboratory-scale milk samples was generated based on a central composite design (CCD) and used to establish partial least squares (PLS) regression for the predictions of sucrose and lactose contents resulting in R^2^ values of 0.9749 and 0.9987 with the corresponding root mean square error of calibration (RMSEC) values of 1.69 and 0.35. However, the physical difference between the laboratory-scale powder and the final product milk tablet samples resulted in spectral deviations that dramatically affected the predictive performance of the PLS models. Therefore, calibration transfer methods called direct standardization (DS) and piecewise direct standardization (PDS) were used to adjust the NIR spectra from the real milk tablet samples before the quantitative prediction. Using high-performance liquid chromatography (HPLC) as a reference method, the developed NIR-chemometric model could be used to instantly predict the sugar contents in real milk tablets by producing root mean square error of prediction (RMSEP) values for sucrose and lactose of 5.04 and 4.22 with Q^2^ values of 0.7973 and 0.9411, respectively, after the PDS transformation.

## Introduction

Milk tablets are considered an alternative nutritious snack. This snack not only delivers a pleasant taste of milk but also is a good source of high-quality nutrients such as protein, carbohydrates, and calcium. Along with milk, sugars are among the major ingredients of milk tablets. Often, sucrose is added to sweeten the milk tablets^1^. Lactose is the main carbohydrate found in raw milk material^2^. Determination of sugar contents is important for quality control evaluation. In addition, variations in the amount of sugars can influence the nutritional quality of the finished product contributing to the eating texture of the milk snack^3,4^.

Traditionally, the quantification of sugar in milk can be achieved using chemical titration methods^5^. These wet laboratory methods can be simple; however, the experimental procedures are time-consuming and often result in large amounts of chemical waste. Several analytical techniques, such as high-performance liquid chromatography (HPLC)^6,7^, electrochemical analysis^8,9^, and nuclear magnetic resonance (NMR)^10^, were also used to accomplish the quantification task. Nevertheless, a significant drawback was that complicated sample preparation methods were required before the sample detection process. Therefore, these tests are not suitable for manufacturing plants where many samples are obtained from a continuous online production process.

Near-infrared (NIR) spectroscopy investigates the vibrational interaction between samples and electromagnetic radiation in a region of 800–2500 nm. NIR detection has several advantages over other spectroscopic detections in that a large number of samples can be non-destructively measured within a short period without extensive sample preparation. The NIR spectra can be related to interesting chemical properties in the samples using a calibration model called partial least squares (PLS) regression^11–13^, where the relationship information between the spectral data and the chemical property is mathematically investigated. This correlation information can then be used to estimate the chemical properties of unknown samples. For example, the NIR spectrometers were recently applied for compositional analysis of cow^14^ and human^15^ milk.

Generally, determinations achieved by calibration models are based on the previous knowledge provided by a set of training samples. Accordingly, variations of the test samples could be estimated by those found in the training set to get the optimal predictive results. Hence, the training samples can significantly characterize the predictive performance of the calibration models. However, in the production process where variations of the final process samples are not expected and should be limited, the final products from the manufacturing process may not contain enough variability to establish a training set for developing accurate and robust calibration models. For example, the training samples in powder form that were prepared in a laboratory room had different physical structures when compared to the final process samples that were eventually pressed into tablets. Sarraguça and Lopes^16^ reported that using laboratory-scale samples in the powder form provided more accurate predictive results than the tablet samples produced by the production process. Additionally, Peerapattana et al.^17^ observed that powder samples of mangosteen pericarp retained in glass vials resulted in better predictive results for the prediction of alpha-mangostin content compared to the samples retained in transparent capsules.

Calibration transfer (CT) involves a group of chemometric methods that can be used to minimize inconsistencies from different instrumental measurements^18^. After establishing the standardization using the CT methods, a system involving the calibrated model can be developed in which one instrument (primary) can be substituted for another instrument (secondary) and vice versa. For instance, a calibration transfer method called piecewise direct standardization (PDS) was used to minimize the variations between a top-bench spectrometer and a portable NIR detector^19,20^. As a result, the estimation of the portable NIR measurement can be based on the data-based NIR spectra collected from the top-bench NIR spectrometer. In addition, by following the same methodology, different variations among agricultural product samples could be identified and systematically discarded from unknown samples for adulteration detection^21^.

This research developed an instant method for the detection of the sugar contents in milk tablets based on the NIR detection. In addition, calibration transfer methods were adopted to adjust the different variations in the NIR spectra between the laboratory-scale milk samples and the milk tablets obtained from the final process. The quantitative analysis was based on multivariate predictions of the PLS models aiming to instantly quantify the concentrations of sucrose and lactose in the milk tablet samples.

## Materials and methods

### Details of milk samples

A total of 13 different milk tablet brands were obtained from local grocery stores in Chiang Mai, Thailand. The relevant details of the milk tablets are summarized in Table 1. The samples were divided into three groups, namely training (T1–T3), internal validation (I1–I3), and external validation (E1–E7) samples. Each milk tablet was ground into a fine and homogeneous powder using a ceramic mortar and pestle. To generate systematic variations representing the sugar contents in the milk samples, a central composite design (CCD) structure was used^22^, comprising nine experiments for each sample. For example, for samples T1, T2, and T3, amounts of sucrose (analytical grade, > 99% purity, RCI Labscan, Bangkok, Thailand) and lactose (analytical grade, > 99% purity, KEMAUS, NSW, Australia) were added to the milk powder according to the coded values of the CCD structure presented in Table 2. Then, a combination of the three CCD model samples was used to construct the training set, which resulted in a total of 27 milk samples. The use of the CCD structure was to ensure that the variation in the recorded NIR spectra was related to the concentrations of the sugars in the milk samples and that the number of training samples was sufficient for establishing the prediction models^23^. Samples I1, I2, and I3 were used to establish internal validation samples, while the variations in the sugar contents were also generated based on the CCD model. Therefore, 27 additional milk powder samples were used to construct the internal validation set. Samples E1–E7 were used as the external samples to represent the independent test set. These were utilized to evaluate the performance of the calibration models when real samples were introduced.

**Table 1:** Concentrations of sucrose and lactose in milk tablet samples.

Sample names	Concentration of sugars (%w/w)	
	Sucrose	Lactose
1. T1 (training)	21.31	36.24
2. T2 (training)	27.11	28.66
3. T3 (training)	14.20	26.46
4. I1 (validation)	27.76	25.71
5. I2 (validation)	27.00	33.40
6. I3 (validation)	22.31	28.48
7. E1 (test)	21.78	38.99
8. E2 (test)	39.04	0.000
9. E3 (test)	43.69	0.000
10. E4 (test)	28.04	28.47
11. E5 (test)	20.06	30.68
12. E6 (test)	16.72	29.32
13. E7 (test)	25.48	28.94

**Table 2:** The CCD structure for generating the training samples.

No. of samples	Code values		Amount of sugar added (%w/w)	
	Sucrose	Lactose	Sucrose	Lactose
1	− 1	− 1	0.000	0.000
2	− 1	0	0.000	15.00
3	− 1	1	0.000	30.00
4	0	− 1	15.00	0.000
5	0	0	15.00	15.00
6	0	1	15.00	30.00
7	1	− 1	30.00	0.000
8	1	0	30.00	15.00
9	1	1	30.00	30.00

It should be noted that two main types of milk tablets were used in this research. Samples E2 and E3 were non-milk-containing tablets or “cheap milk tablets” where artificial milk flavor was added to achieve product satisfaction. On the other hand, the rest of the milk samples were produced from cow’s milk as raw material and were referred to as “premium milk tablets”.

### NIR spectral detection

The NIR spectra of the milk powder (9.00 g) were acquired using a NIR transportation module (width × length × depth: 5.7 × 29.4 × 2.0 cm) equipped with the NIRSystem 6500 (Multi-Mode™ Analyzer, Foss, USA) in the range of 400–2500 nm at a 2 nm sampling interval, yielding 1050 data points per spectrum. An average of 64 scans was used for each sample. The samples of the milk tablets were placed inside the NIR transportation module. Layers of the milk tablets were directly attached to the containing glass following the measurement conditions of the powder samples. The milk samples were maintained at a room-controlled temperature of 25 °C for at least 6 h before the NIR detection. Prior to the analysis, the NIR spectra were pretreated by standard normal variate (SNV) to eliminate errors caused by the light scattering during the NIR measurement. Then, they were mean-centered so that the analysis focused on the variance from the data mean rather than the absolute values.

### HPLC analysis of sugar determination

Sugar contents in the milk tablet samples were measured using high-performance liquid chromatography (HPLC). For sample preparation, 1.00 g of each ground milk tablet was dissolved in 10 mL ultra-pure water and kept in a water bath (Julabo Labortechnik GMbH, Seelbach, Germany) at 55 °C for 5 min. Then, HPLC-grade acetonitrile was added for protein precipitation^24,25^. After the denaturation, the sample solution was centrifuged at 10,000 rpm for 5 min. The clear solution was then filtered through a 0.45 µm nylon syringe filter (Agilent Technologies, CA, USA).

The chromatographic analysis of sugar contents in the milk tablets was carried out with a high-performance liquid chromatograph (Agilent 1100 HPLC system, CA, USA) with an Agilent ZORBAX NH_2_ column (5 µm, 4.6 mm inner diameter, 150 mm length) operating at 25 °C. The samples were auto-injected into the HPLC system with an injection volume of 10 µL. A mixture of HPLC-grade acetonitrile and ultra-pure water (75/25%v/v) was used as a mobile phase with a flow rate of 1.00 mL/min. A refractive index detector (RID) was operated at 25 °C. The sugar contents were determined using the external standard calibration curve of the sucrose and lactose standards resulting in R^2^ values of 0.9907 and 0.9896, respectively. The concentration values of the sugars in the studied milk tablet samples are summarized in Table 1.

### Chemometric analysis

#### Standardization of NIR spectra using DS and PDS calibration transfers

Although both forms of the milk samples (tablet and powder) were considered solid, there were differences, for example, in particle size and tablet compaction pressure. These physical variations resulted in significant deviations in the recorded NIR spectra^26^. Calibration transfers are multivariate correction methods that can be applied to stabilize variations that may have occurred due to differing instrumental and measurement conditions. In this research, they were used to account for any signal discrepancies between the spectra obtained from the tablet samples and the powder samples. Piecewise direct standardization (PDS) is an extension algorithm of a conventional method called direct standardization DS^27,28^. The DS method describes the correlation between the two data matrices (***X***_*m*_ and ***X***_*s*_ referring to master and slave data) by calculating a transformation matrix (***F***) using multiple linear regression models such as MLR, PCR, and PLS:

$${\varvec{X}}_{m} = {\varvec{X}}_{s} \times {\varvec{F}}$$

The extension in the PDS algorithm is that each spectral point of the master data (***X***_*m*,*j*_) is specifically related to a spectral subset of the slave data (***X***_*s,j*_). The PDS algorithm involves the following steps:

- Step 1: Select the spectral points of the master data (***X***_*m,j*_) at wavelength *j.*
- Step 2: Define the subset spectra of the slave data (***X***_*s,j*_) nearby wavelength *j* to form index *j* − *k* to *j* + *k*
  $${\varvec{X}}_{s,j} { = [}{\varvec{x}}_{s,j - k} \cdot {\varvec{x}}_{s,j - k + 1} {, } \ldots {, }{\varvec{x}}_{s,j + k - 1} \cdot {\varvec{x}}_{s,j + k} ]$$
  where *k* is the window size controlling the amount of the spectral data which will be used in the calculation.
- Step 3: Establish the regression coefficient
  $${\varvec{X}}_{m,j} = {\varvec{X}}_{s,j} \times {\varvec{b}}_{j}$$
  where ***b***_i_ is a vector containing regression coefficients.
- Step 4: Generate the transformation matrix (***F***) by organizing the ***b***_*j*_ into a diagonal matrix
  $$\varvec{F }={\text{diag}}\;{(}{\varvec{b}}_{{1}}^{T} {,}\;{\varvec{b}}_{{2}}^{T} {,} \ldots \;{\varvec{b}}_{j}^{T} {,} \ldots \;{\text{b}}_{n}^{T} )$$
  where *n* is the number of spectral channels included.
- Step 5: Standardize the spectra of unknown samples (***X***_*s,un*_) using ***F*** to obtain the modified spectrum (***X***_*s,PDS*_)
  $${\varvec{X}}_{s,PDS} = {\varvec{F}} \times {\varvec{X}}_{s,un}$$

In this research, the DS and PDS transformations were used to account for the inconsistencies between the spectra obtained from the powder samples and the tablet samples. These transformation methods investigated the correlation between the two datasets. After that, the resulting correlation information was applied to adjust the NIR spectra of the milk tablet samples. Consequently, the adjusted data could be compatible when making the prediction using the calibration model established from the NIR spectra of the powder samples without the need to recalibrate the model.

The model optimization was based on a previously published report^21^. The correlation matrices in both DS and PLS were determined using PLS regression which was calculated using the training samples and optimized based on the internal validation samples.

#### PLS for quantitative analyses

Partial least squares (PLS) regression is among the most potent analysis methods of multivariate calibration models^29^. The significant advantage of the PLS algorithm is that the variations obtained from both the predictive and response parameters are simultaneously extracted and then used to construct the prediction model. With the use of the PLS model, the correlation between these information blocks could be maximized. In most cases, PLS could successfully offer the optimal predictive performance for the prediction of the NIR spectral data^11,30^.

In this research, the NIR spectra and the sugar contents were, respectively, used as predictive and response parameters for the PLS models. The PLS calculation was done following the procedure described in the previously published literature^29^. The leave-one-out cross-validation method was applied to identify the optimal number of PLS latent variables^31^. According to Table 1, PLS models were developed using training (T1–T3) samples as calibration data. To validate the models, internal validation (I1–I3) and external validation (E1–E7) samples were used for validation and prediction, respectively.

The predictive performance of the PLS models in terms of prediction accuracy was reported by root mean square error of calibration (RMSEC) and root mean square error of prediction (RMSEP). The coefficients of determination for the calibration (R^2^) and prediction (Q^2^) values were calculated to determine the robustness of the models. In addition, the standard error of the cross-validation (SECV) and the ratio of prediction to deviation (RPD) were employed to compare the different predictive performances of the calibration models^32^. The calculations of the PLS model, PDS calibration transfer, and statistical analyses were implemented using in-house MATLAB scripts (MATLAB, The Math Works Inc., Natick).

## Results and discussion

### NIR detection

#### Exploratory data analysis of NIR spectra data

**Figure 1:**
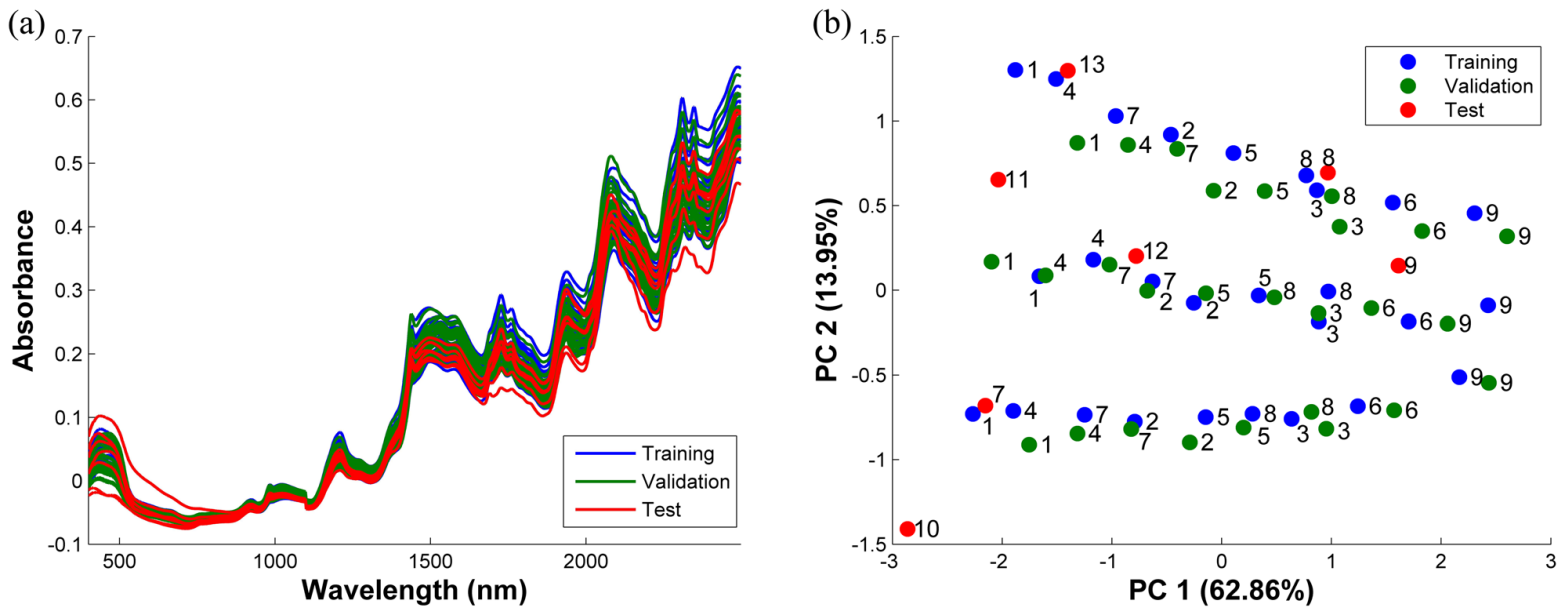
(**a**) NIR spectra of the studied milk samples (powder) and (**b**) PCA score plot of the corresponding NIR spectral data.

Figure 1A illustrates the NIR spectra recorded from the milk samples. The corresponding PCA score plot of the NIR spectra was generated to demonstrate the characteristic differences among the milk samples presented in Fig. 1b. In this research, the training and validation samples were generated based on the CCD experiments to induce systematic variations due to the sugar concentrations. In the PCA score plot, the samples were scattered across the PCA space, wherein a greater PC1 value represented the sample with high sucrose content. On the other hand, higher PC2 values were associated with the samples with high lactose content.

Consequently, it was clear that the sample organizations were achieved due to the variations in the sugar contents. This demonstrated that the NIR detection could analyze the milk samples with different sugar contents. Most of the test samples were placed within the region of the training samples implying the suitability of the generated training samples. Test sample no. 10 (E10) was located slightly away from the main cluster; however, this could be due to the extracted malt, which was utilized as a flavoring agent and resulted in yellow–brown double-layer tablets.

#### NIR spectral transformation of milk tablet samples

**Figure 2:**
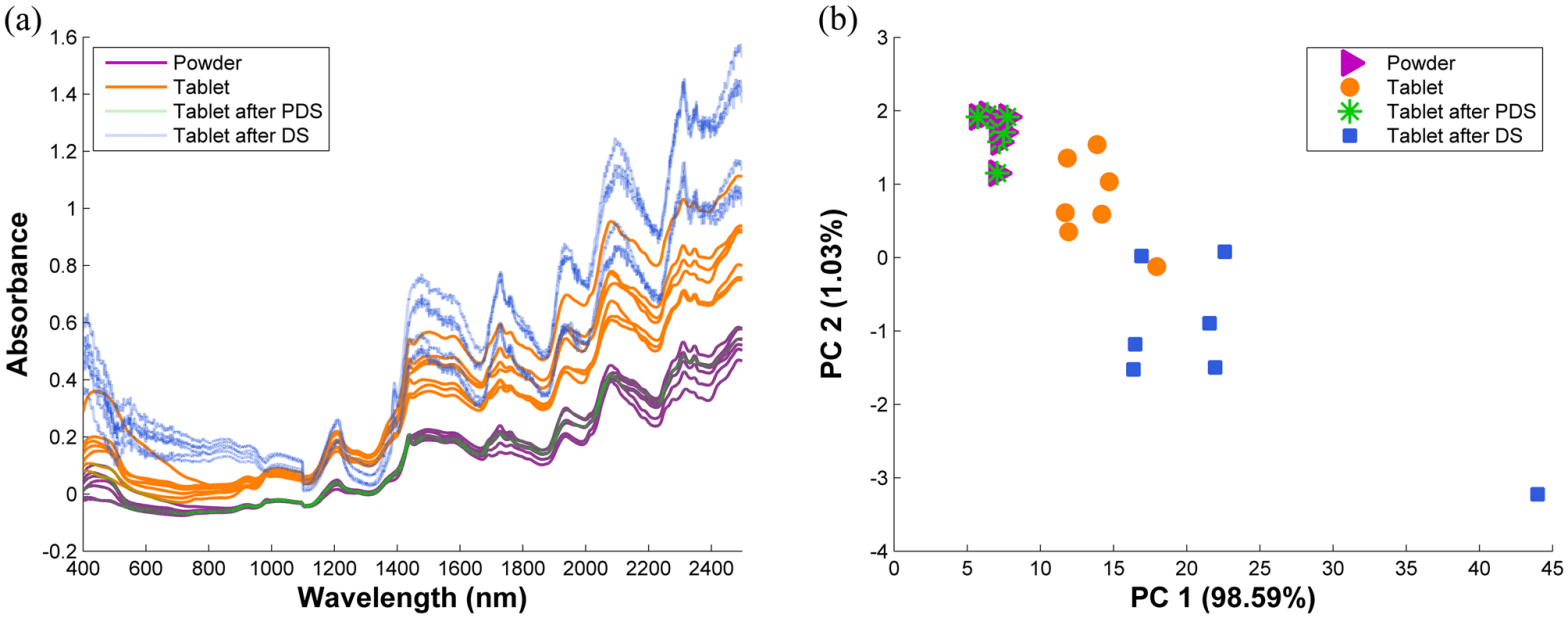
(**a**) NIR spectra of the test milk samples (powder and tablet) and (**b**) PCA score plot of the milk samples after the DS and PDS transformations.

Figure 2 presents the difference in the NIR spectra between the powder and tablet milk samples. The NIR spectra of milk tablets (E1–E7) were recorded before and after grinding into powder. In Fig. 2a, the shapes of the NIR spectra obtained from both physical states were relatively similar. However, the powered milk samples resulted in relatively lower levels of absorbance, which could be related to the particle size of the samples. This outcome corresponded to the finding of a previous report noting that tablet compaction pressure resulted in more intense penetration with higher absorbance intensity of the NIR spectra^26^. The variation that occurred because of the sample's physical conditions could be confirmed in the PCA score plot shown in Fig. 2b. From the score plot, the milk samples without any transformation process were separately clustered into two main groups, wherein the tablet and powder samples were placed differently on the PCA space. The clear separation between the sample clusters implied that the physical differences caused more variation than chemical compositional differences between milk samples. In other words, in this experiment with the PCA visualization, the physical variation identified between the powder and the tablet samples could be systematically captured by the first two PCs.

After the spectral data recorded from the tablet samples were transformed using the PDS transformation process, the shape of the spectra was significantly changed and became very similar to that of the powder samples, as shown in Fig. 2a. The success of the spectral transformation can be confirmed by the PCA score plots shown in Fig. 2b, in which the transformed samples were placed nearly exactly in the identical PCA locations as the powder samples. In this case, PDS transformation could effectively remove the variation that occurred due to the physical state of the samples. Commonly, PDS transformation is used to adjust for any differences in the experimental conditions; for example, the difference observed in the sample detection methods using two different instruments, namely primary and secondary. The correlation between the instruments was investigated based on the PLS-NIPAL algorithm during the modeling process^33^. As a result, systematic variations in the spectral data obtained from both instruments were extracted concurrently, while also maximizing their covariance values. This correlation information could be used to transform the detection signal from the secondary instrument with respect to the covariation structure of both instruments. Consequently, the transformed signals could be well-suited to the prediction models established from the primary instrument without requiring the prediction model to be regenerated. In this experiment, the reconstruction of the transformed NIR spectra was based on the systematic structure, which could be effectively captured by the PCA modeling, as previously discussed. Therefore, PDS could be successfully adapted to the translation of the NIR spectral data of the tablet samples. These results indicated the effective utilization of the PDS transformation model for stabilizing the NIR spectra recorded from samples with different physical appearances.

In this research, the DS adjustment which was based on a single calculation of the correlation matrix produced unsatisfactory estimates of the NIR spectra. However, the visualization of this spectral transformation was based on the use of raw NIR spectral data without data pretreatment. This global adjustment using DS could be sensitive to the drift in the spectral baseline as visualized in Fig. 2a and this demonstrated that the calculation based on the spectral subsection could improve the performance of the calibration transfer process. The effect of this data adjustment on the prediction performance will be discussed in the prediction of the sugar content using the PLS model.

### Detection of sugar contents using PLS

#### Prediction of internal validation samples

The NIR spectra of the training samples were used to establish PLS calibration models to estimate the sugar contents in the milk samples. Using the PLS1 algorithm, the calibration model was constructed independently to predict each type of sugar. The validation samples were used to investigate the predictive performance of the developed PLS models, and the predictive results were summarized in Table 3.

**Table 3:** Statistical values for the PLS prediction using the training and internal validation milk samples.

Model statistics	Sucrose	Lactose
R^2^	0.9749	0.9987
Q^2^	0.9373	0.9943
RMSEC	1.69	0.35
RMSEP	2.67	0.79
SECV	3.28	5.02
RPD	4.09	12.48

**Figure 3:**
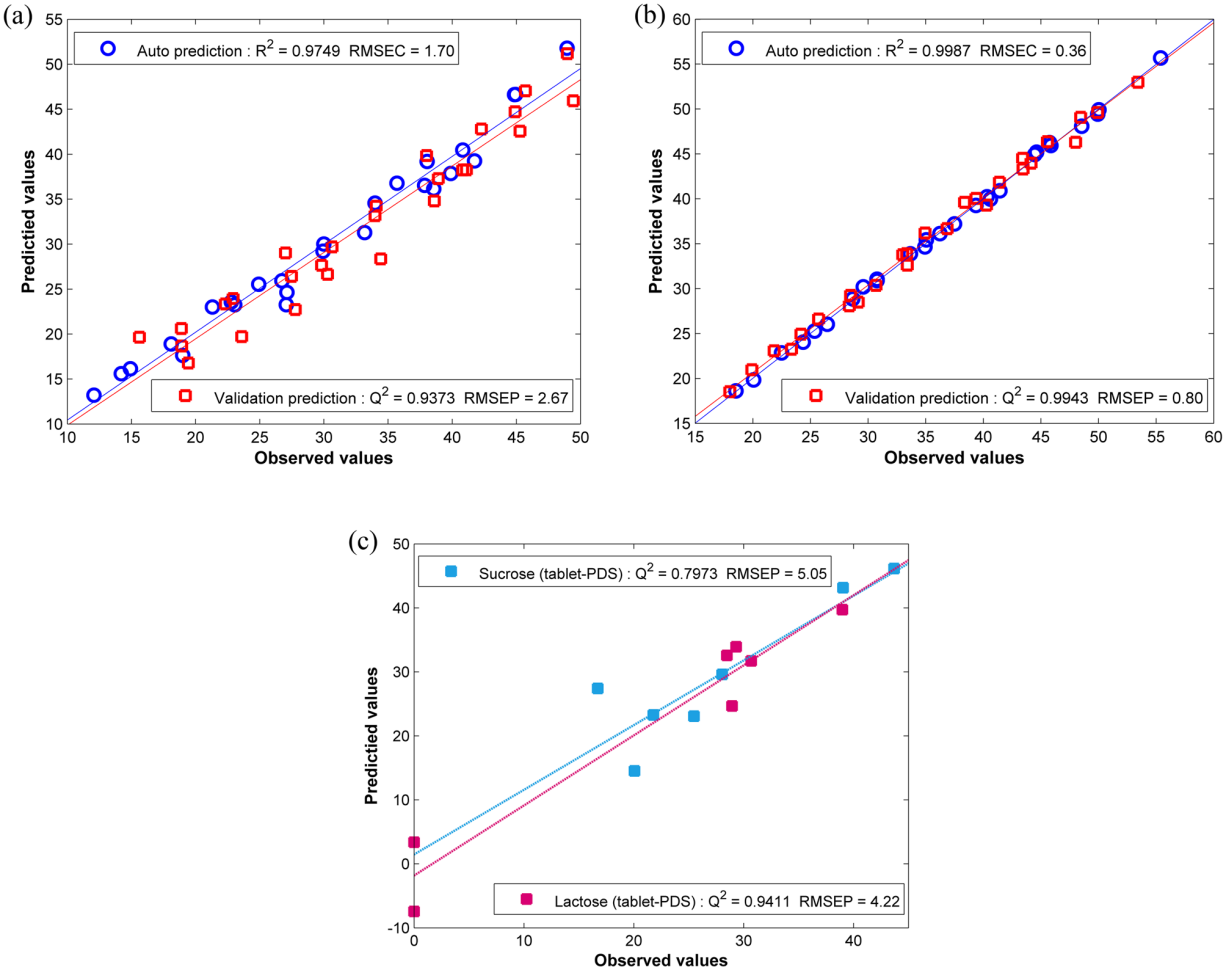
Correlation graphs for the training and internal validation samples of (**a**) sucrose, (**b**) lactose contents, and (**c**) the real milk tablet samples after PDS transformation for the predictions of the sucrose and lactose contents.

The PLS model for the prediction of the sucrose content resulted in calibration performance values for RMSEC, RMSEP, R^2^, and Q^2^ of 1.69, 2.67, 0.9749, and 0.9373, respectively. At the same time, the prediction of the lactose content generated predictive results for RMSEC, RMSEP, R^2^, and Q^2^ values of 0.35, 0.79, 0.9987, and 0.9943, respectively. A relatively small value of RMSECs implied that the PLS successfully fitted the data and that the calibration models adequately modeled the response parameter variations. The PLS models could generate high R^2^ and Q^2^ values implying that the developed models were stable and could successfully be used to estimate the sugar contents in the prepared powder samples. These results corresponded to the correlation graphs presented in Fig. 3a,b, where the predicted samples were placed approximately close to the diagonal lines of the graphs suggesting that most of the samples had slight differences between the reference and the predicted sugar concentrations. The values of Q^2^ were slightly lower than the R^2^ values implying that the models were not prone to the overfitting problem.

In general, RPD values allow for a comparison of the prediction accuracy of the different models. Higher values of RPD would indicate a better predictive ability corresponding to lower bias and SECV values along with higher Q^2^ values. The established model for lactose prediction had a higher RPD value than the prediction of sucrose, indicating that the PLS model had a better predictive performance. These findings suggest that lactose, a naturally occurring sugar in milk, could be used to obtain additional related information from other components, such as the fats and proteins presented in the raw-milk material. In comparison to the sugar naturally found in food products, sucrose, which was added during the preparation process, contained less micronutrient information^34^.

#### Prediction of sugar content in real milk tablet samples

Table 4 shows the predictive results of the milk tablets (E1–E7) using the developed PLS models. When the developed PLS models were used to estimate the sugar contents from the NIR spectra directly detected from the tablet samples (P to predict T), higher predictive errors were obtained, resulting in significantly lower Q^2^ values (0.7024 and 0.7030 for sucrose and lactose, respectively) and higher RMSEP values (53.44 and 8.14 for sucrose and lactose). The poor predictive results correspond with the PCA score plot shown in Fig. 2b, where the deviations between the powder and tablet samples were highlighted, noting that the physical structures of the samples could significantly affect the NIR measurements.

**Table 4:** Sugar determinations in real milk tablet samples.

Sample names	Sucrose amount (%w/w)				Lactose amount (%w/w)			
	Actual	P predict T***	P* predict T/P** (DS)	P* predict T/P** (PDS)	Actual	P predict T***	P* predict T/P** (DS)	P* predict T/P** (PDS)
1. E1	21.78	82.31	41.68	23.27	38.99	33.14	18.16	39.72
2. E2	39.04	84.36	58.79	43.15	0.00	− 2.351	41.41	− 7.442
3. E3	43.69	95.56	45.36	46.15	0.00	12.14	− 13.70	3.382
4. E4	28.04	76.37	39.66	29.60	28.47	43.15	16.65	32.57
5. E5	20.06	72.33	35.11	14.52	30.68	26.10	13.41	31.72
6. E6	16.72	76.17	42.41	27.41	29.32	29.77	11.46	33.92
7. E7	25.48	80.08	53.56	23.07	28.94	22.54	− 0.2068	24.66
	Q^2^	0.7024	0.2853	0.7973	Q^2^	0.7030	0.0002	0.9411
	RMSEP	53.44	19.27	5.04	RMSEP	8.14	23.73	4.22

*P = powder samples, **T/P = tablet samples adjusted to powder samples, ***T = tablet samples.

**Figure 4:**
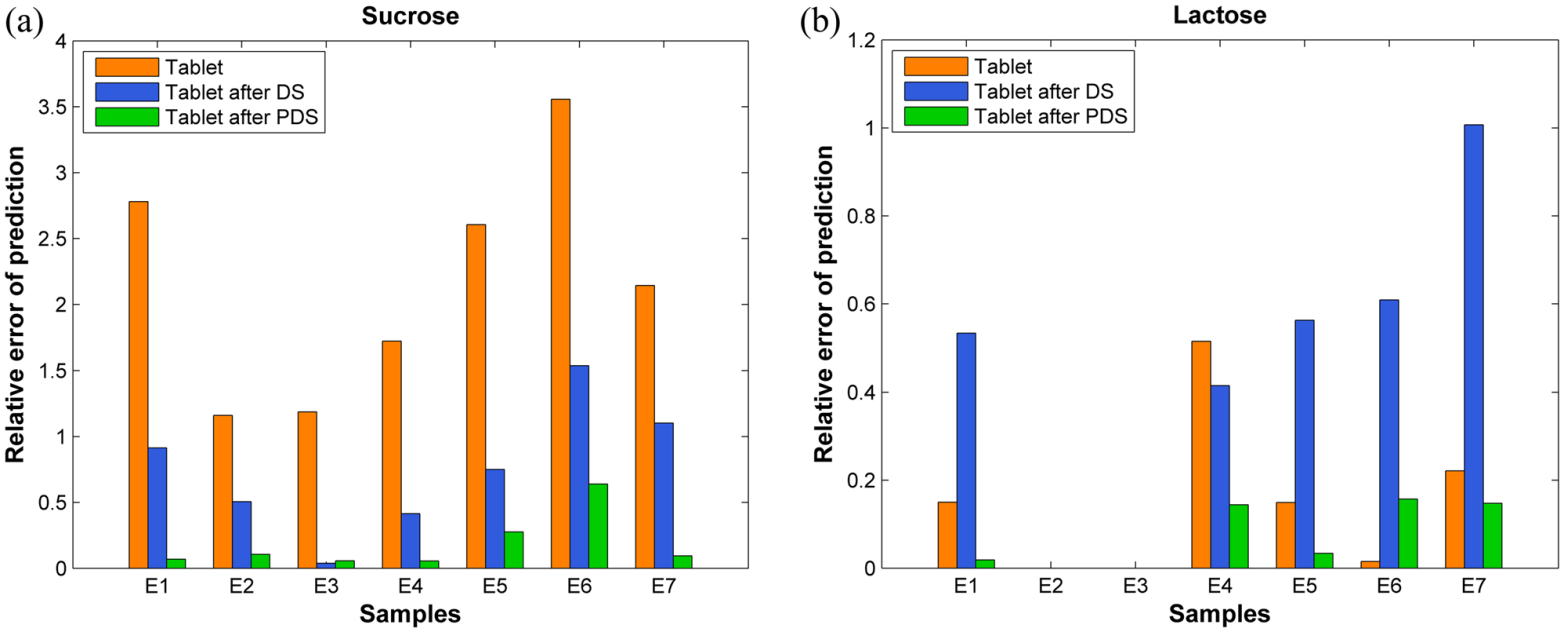
Comparison between the prediction results before and after the DS and PDS transformations of the real milk tablets.

Significant improvements in the predictive results could be obtained after the NIR spectral data were preprocessed by PDS transformation (P to predict T/P). Figure 4 illustrates the comparison between the predictive results before and after the PDS transformation demonstrating the significant reduction in the relative errors associated with non-destructive detection. In Table 4, the Q^2^ values for both sucrose and lactose prediction models increased to 0.7973 and 0.9411, respectively. The corresponding correlation graphs of the reference and the predicted sugar contents for the PLS models are shown in Fig. 3c. The DS transformation resulted in the improvement in the prediction of the sucrose content having a reduction of the RMSEP value to 19.27, but it failed to improve the results for the prediction of the lactose content where the RMSEP was increased to 23.73. This confirmed that the calculation based on the spectral subset in the PDS method could improve the predictive accuracy of the PLS calibration models.

The lactose concentrations in samples 8 and 9 were null because they were milk tablets that contained only artificial-milk flavor without the actual cow’s milk component. These results indicated that the laboratory-developed model could be used to predict real milk tablet samples or the final process samples by enabling uncomplicated measurements and real-time analysis of a large number of samples collected during the manufacturing process without the need for sample preparation.

## Conclusion

In the manufacturing process, it was not practical to produce modeling samples that had enough variations to generate robust prediction models. In addition, the spectral deviations that occurred while completing the NIR measurements could have affected the prediction accuracy of the developed model. This research demonstrated that using the calibration transfer method widened the utilization ability of the developed calibration models. NIR spectroscopy combined with chemometric analyses can be applied to detect the sugar contents in milk tablets. PDS resulted in an improved level of the predictive performance of the tablet samples. The development process offered non-destructive, accurate, and rapid techniques for determining sugar contents in real milk tablet samples.

## Data Availability

The NIR spectroscopic datasets analyzed during the current study are available from the corresponding author upon reasonable request.
